# Independent regulation of *Plasmodium falciparum rif* gene promoters

**DOI:** 10.1038/s41598-018-27646-0

**Published:** 2018-06-19

**Authors:** Rosana Beatriz Duque Araujo, Tatiane Macedo Silva, Charlotte Sophie Kaiser, Gabriela Fernandes Leite, Diego Alonso, Paulo Eduardo Martins Ribolla, Gerhard Wunderlich

**Affiliations:** 1 Department of Parasitology, Institute of Biomedical Sciences, University of São Paulo, Av. Prof. Lineu Prestes, 1374, São Paulo - SP, 05508000 Brazil; 2 Institute of Animal Physiology, Schloßplatz 8, Westfälische Wilhelms Universität, Münster, Germany; 3 Department of Parasitology, IBB/IBTEC, State University of São Paulo, Botucatu, São Paulo Brazil

## Abstract

All *Plasmodium* species express variant antigens which may mediate immune escape in the vertebrate host. In *Plasmodium falciparum*, the *rif* gene family encodes variant antigens which are partly exposed on the infected red blood cell surface and may function as virulence factors. Not all *rif* genes are expressed at the same time and it is unclear what controls *rif* gene expression. In this work, we addressed global *rif* transcription using plasmid vectors with two drug resistance markers, one controlled by a *rif* 5′ upstream region and the second by a constitutively active promoter. After spontaneous integration into the genome of one construct, we observed that the resistance marker controlled by the *rif* 5′ upstream region was expressed dependent on the applied drug pressure. Then, the global transcription of *rif* genes in these transfectants was compared in the presence or absence of drugs. The relative transcript quantities of all *rif* loci did not change profoundly between strains grown with or without drug. We conclude that either there is no crosstalk between *rif* loci or that the elusive system of allelic exclusion of *rif* gene transcription is not controlled by their 5′ upstream region alone.

## Introduction

The infection with one of the five *Plasmodium* species which cause malaria in humans still is a challenge for the public health predominantly in underdeveloped countries. *Plasmodium falciparum* alone is still responsible for 445000 deaths per year, mostly children under five years or pregnant women^1^. Part of the virulence exerted by *Plasmodium* is caused by the presence of variant antigens expressed on the surface of the host’s infected red blood cells. Members of the best-characterized family of variant antigens, the *P. falciparum* erythrocyte membrane protein 1 (PfEMP1), play a central role in immune evasion. PfEMP1 are encoded by approximately 50–60 different *var* genes^2^ which are highly recombinogenic^3,4^, possibly through specific three-dimensional DNA conformations near breakpoints^5^, and possess a modular structure^6^. In order to successfully evade immune mechanisms exerted by the human host, *var* gene transcription is tightly controlled in a way that normally only one or two *var* genes are expressed. A number of factors are involved in this control and these include not only sequences in the 5′ upstream regions of *var* genes^7,8^, including untranslated ORFs^9,10^, the pairing of *var* promoters and *var* introns^11^, but also specific DNA/chromatin binding factors^12^ and the involvement of several chromatin modifiers (reviewed in^13^). Recently, the participation of non-coding GC-rich RNAs in *var* transcription control was shown^14^. The current model of *var* transcription regulation also suggests a specific subnuclear site in which *var* transcription occurs and to which *var* loci translocate in order to be transcribed. However, the exact factors and dynamics which orchestrate this process and license one *var* locus for transcription while excluding all other *var* loci are largely unknown. Still more elusive is what determines that a *var* locus and its associated histone modifications switch from an active to a silent state or vice versa.

Another major variant gene family which is not only found in human or primate *Plasmodium* species but also in murine species is the *pir* (*Plasmodium* interspersed repeat) gene family^15^, and the biological function of gene products from this family is not well understood. Recent results indicate that their encoded proteins may function at different points of the parasite-host interface^16^. If results from *Plasmodium chabaudi* can be extrapolated to all *Plasmodium* species, *pir* transcription seems to be reset during mosquito passage and reinfection^17^ and earlier evidence pointed to transcriptional diversity^18^ and quick switching in several models^19,20^. A recent study implied a specific PIR protein of *P. falciparum* (termed RIFINs, repetitive interspersed family^21^) as a factor involved in the pathogenic process of erythrocyte rosetting^22^. Another study revealed that a specific motif in RIFINs promoted binding to leucocyte immunoglobulin-like receptor B1 (LILRB1) or leucocyte-associated immunoglobulin-like receptor 1, thereby inhibiting activation of B-cells and natural killer cells which express the LILRB1 receptor^23^. This turns evident that at least some RIFINs can be understood as virulence factors. Importantly, VIR proteins of *Plasmodium vivax* also appear to mediate cytoadherence and participate in pathogenic processes and probably immune evasion^24,25^. In *P. falciparum*, the vast majority of *rif* genes are localized adjacent to *var* genes, often in a tandem organization. In version 36 of PlasmoDB, there are 221 genes in the 3D7 strain genome which encode PIR proteins. Of these, 158 are full-length RIFINs, 27 are truncated or defect RIFINs, and the remaining are STEVOR (**s**ub**te**lomeric **v**ariant **o**pen **r**eading frame) or truncated or defect STEVOR. RIFINs can be categorized into two major groups of *rif* genes and RIFINs: A and B. These groups differ by a short conserved 25mer peptide sequence in the first half of the protein which is present only in the 97 A-group RIFINs in the *P. falciparum* strain 3D7 genome^26^. While the A-group RIFINs seem to be exported to the infected red blood cell (IRBC) surface, B-group RIFINs are believed to remain associated with the vesicular network in the IRBC (Maurer’s clefts). It is still unclear what controls *rif* transcription, a recent study pointed to the transcription factor AP2-SP^27^ which somehow seems to influence transcription in blood stage *P. falciparum*^28^. While earlier studies indicated that *rif* and *var* gene transcription may be controlled by similar factors^29^, no clear-cut allelic exclusion mechanism could be detected in other studies which specifically addressed *rif* transcription or switching^20,30^. A drawback in the study from Howitt and colleagues^29^ was that the tested *rif* promoter controlled the unique drug resistance marker blasticidin deaminase in their construct, turning a considerable baseline activity of the promoter essential in order to obtain transfectant parasite lines. This did not permit a genuinely “switched-off” state of the *rif* 5′ ups-controlled transgene. In another study, the activity profile of a 5′-*rif* upstream region appeared more related to *var* genes^31^ and no significantly different regulation could be discerned upon activation. Also, no “crosstalk” – understood as the influence of the activity of one promoter on the activity of remaining promoters - between 5′ upstream regions such as occurs in *var* gene regulation was detectable. In contrast, in the study by Goel and colleagues, phenotypic selection procedures pointed to the expression of a single *rif* gene in parasites with PfEMP1-independent rosetting^22^, supporting the view that allelic exclusion and crosstalk may occur. In order to settle the question if there are allelic exclusion and crosstalk between *rif* 5′ ups regions, we used three different *rif* 5′ upstream regions in bicistronic transfection plasmids. Previously, this approach was successfully applied on *var* promoters^7,32^. After transfection, we divided transfected parasite lines and in one culture we selected the growth of parasites which actively transcribed a drug resistance marker (human dihydrofolate reductase). Afterwards, we compared *rif* transcripts between pyrimethamine-derivate sensitive and resistant lines by RNAseq.

## Results

**Figure 1:**
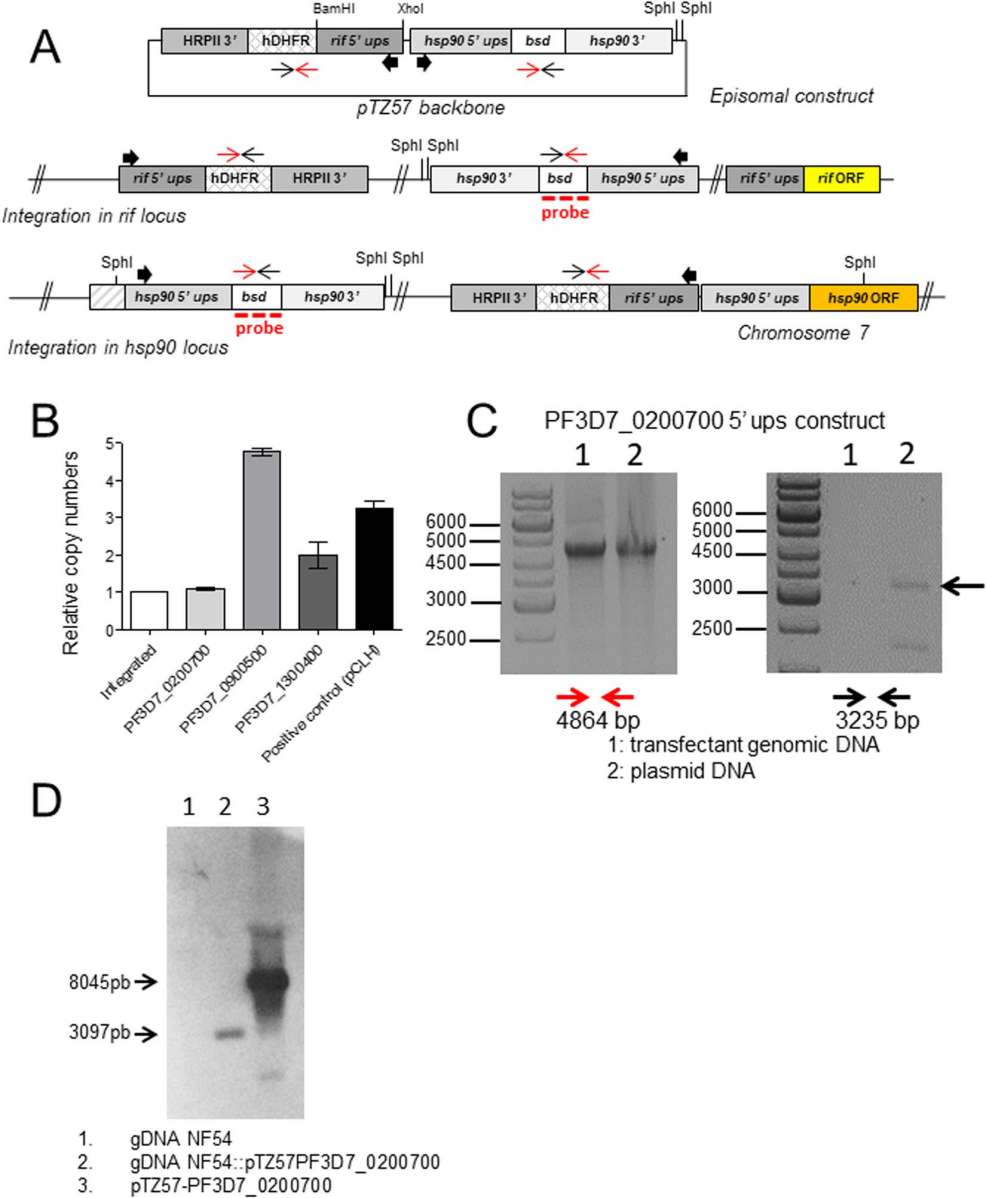
Only the PF3D7_0200700 construct shows single integration in the hsp90 locus. In (**A**) the outline of the transfection plasmid constructs is shown. *rif* 5′ ups regions were interchanged in this plasmid by ligation of XhoI/BamHI fragments representing the *rif* 5′ ups sequence. See Material and Methods for oligonucleotide sequences used for amplification of *rif* 5′ ups. Due to identity with *P. falciparum* genomic sequences, Integration may occur in 4 possible sites: hsp90 5′ ups and 3′ downstream regions, in the hrp3 3′ downstream region and in *rif* 5′ ups regions. Due to the size of identical stretches, we focused on the possible integration events in hsp90 5′ ups and *rif* 5′ ups. Bold arrows indicate the expected direction of transcription in the 5′-ups regions. (**B**) Copy number comparison of different transfected parasite lines with gDNA from the integrated NF54::RESA-GFP strain^54^, an episomal pCLH NF54 strain, and the parasite lines after transfection with plasmids containing different 5′ *rif* ups. The primer performance of hDHFR primers and for the t-seryl RNA synthetase were tested and judged identical (less than 0.5 Ct difference on the same substrate in qPCR using gDNA from the NF54::RESA-GFP strain). Then, copy numbers of the hDHFR locus in relation to the genomic t-seryl RNA synthetase were calculated. In (**C**) results from PCRs using long-range polymerases provide indirect evidence that the PF3D7_0200700 5′ *rif* ups was integrated in the genome. On the left, PCR results showing amplification with forward *hDHFR* and forward *bsd* primers (in red) over the backbone of the transfection plasmid (positive for the untransfected plasmid (2) as well as the gDNA from the PF3D7_0200700 5′ *rif* ups transfected strain). On the right, amplification products from PCRs using reverse *hDHFR* and reverse *bsd* oligos. This amplification is only possible when the plasmid is in the episomal form (see scheme for episomal construct and integrated locus in **A**). No amplification product is seen for the gDNA from the strain with the PF3D7_0200700 5′ *rif* ups. See Supplementary Figure 2 for results with the other *rif* ups constructs. In (**D**), digestion with SpHI of NF54 genomic DNA, transfectant line genomic DNA and transfected plasmid DNA and subsequent Southern blot analysis with a probe consisting of a digoxigenin-labeled bsd fragment. Note that only in the case of integration in the hsp90 locus a 3097 bp fragment is formed, while linearized or concatemerized plasmids will result in 8045 bp fragments.

The expression mode of *rif* genes and the proteins they encode, RIFINs, is unclear. In order to test if *rif* genes are expressed in a similarly coordinated way as *var* genes^33^, we created bicistronic plasmids similar to those used by Voss^7^ or Witmer^31^ and colleagues. In these, one resistance marker gene was controlled by the constitutive plasmodial heat shock protein 86/90 promoter and the other by potentially inducible *rif* 5′ upstream sequences. We chose three *rif* upstream sequences (ups) based on previous observations using wild-type parasites of the 3D7 lineage where *rif* transcripts from these 5′ upstream regions were detected^20^. This assured that functional promoters were being used. The size (~1500 nt) of the inserted putative promoter sequence was chosen observing the distance to adjacent ORFs (Supplemental Fig. 1). In Fig. 1, an outline of the plasmid constructs is shown. Plasmids were transfected and blasticidin-resistant parasite lines were readily established. It is possible that transcription of episomal 5′ upstream sequences is different from genomic loci, due to an increase in the number of circulating episomes (for example^31^). When testing the copy number of the hDHFR gene, we observed that the transfectant line containing the PF3D7_0200700 5′ *rif* ups showed only one copy. In contrast, the hDHFR gene in a freshly transfected parasite line containing the *Photinus* luciferase encoding plasmid pCLH (a derivate of pDC10^34^ where the chloramphenicol acetyltransferase was changed for a luciferase coding sequence from pGL2 (Promega)) appeared in approximately three copies per cell (selection using 2.5 nM WR99210, Fig. 1). Also, the transfectant lines containing the PF3D7_0900500 or the PF3D7_1300400 5′ *rif* ups showed five or two hDHFR copies per parasite genome, respectively (Fig. 1). The appearance of only one copy hints to the integration of the bicistronic plasmid. To verify this, we used two PCR amplifications to specifically detect integrated or episomal forms of plasmids in all transfectant lines containing *rif* 5′ ups. As shown in Fig. 1, no amplicon was detected using an oligo pair which amplifies over the putative breakpoint upon single crossover integration of the construct carrying PF3D7_0200700 5′ *rif* ups. On the other hand, an amplicon representing the plasmid backbone both in integrated or episomal forms was detected in both transfectants genomic DNA and purified plasmid controls. The transfectants with the PF3D7_0900500 or the PF3D7_1300400 5′ *rif* ups showed each several amplicons, one of which consistent with episomal forms of the plasmids (Supplementary Fig. 2). Since the transfection plasmid backbone contains four sequence regions which may provoke single crossover recombination into the genome (the hrp3 terminator, the hsp86/90 5′ ups and the hsp86/90 3′ region plus the *rif* 5′ ups region), we conducted a Southern blot analysis using a digoxigenin-labeled bsd-gene fragment as a probe. The observed pattern indicated that recombination occurred at the hsp86/90 locus and not at the PF3D7_0200700 *rif* 5′ locus (Fig. 1).

**Figure 2:**
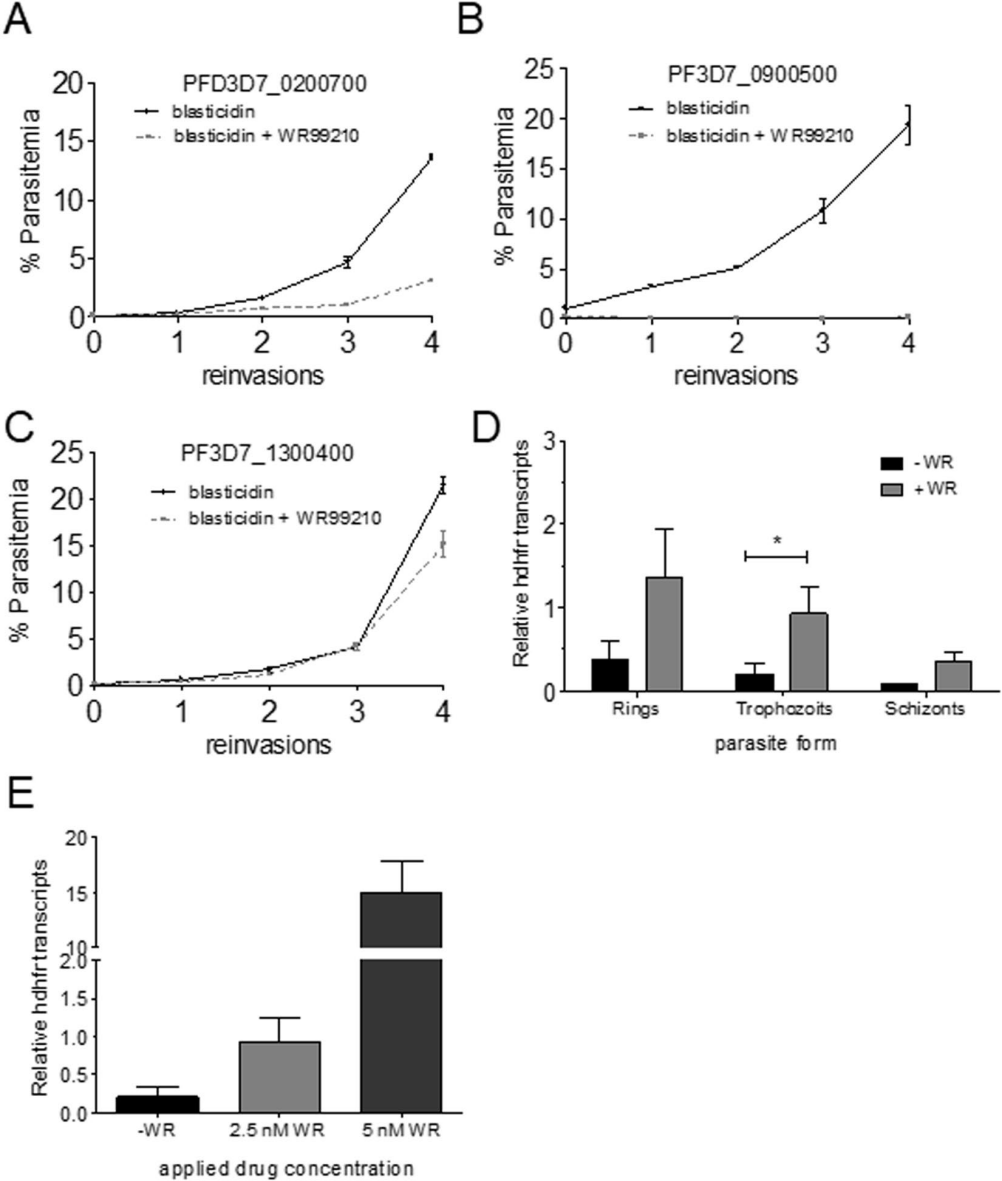
Differential growth of strains containing different 5′ rif ups controlling hDHFR mediated resistance. In (**A**–**C**), transfected NF54 parasite strains with plasmids containing the indicated 5′ *rif* ups controlling hDFHR transcription, established using blasticidin at 2.5 µM as described in methods, were synchronized with plasmagel flotation and sorbitol treatment and submitted to additional WR99210 treatment (2.5 nM) or not. Parasitemias were observed every 48 h hours briefly after reinvasion. In (**D**) the strain with the PF3D7_0200700 5′ *rif* ups construct was analyzed for hDHFR transcript abundance dependent on the blood stage form. This experiment was done in three biological replicates and data are shown. In (**E**) the same trophozoite stage parasites as in (**D)** were submitted to higher concentrations of WR99210 and the hDHFR transcript was measured by RT-qPCR. Error bars in all graphs show standard deviation. Statistical differences between relative transcript quantity values were calculated using the two-way ANOVA test (*p < 0.05).

We then tested if transfectant parasites were able to survive in the presence of the second drug WR99210. A prerequisite for survival is the sufficient transcription from the cloned 5′ *rif* ups. To test this, the cultures of the three parasite lines were split and one half of each culture was cultivated in the presence of 2.5 µg/ml blasticidin and 2.5 nM WR99210, while the other half was solely grown in the presence of 2.5 µg/ml blasticidin. The parasite line with PF3D7_0200700 5′ *rif* ups grew slowly for the first few cycles and then proliferated normally, in accordance with a selection of parasites which showed upregulation of the hDHFR controlling PF3D7_0200700 5′ *rif* ups (Fig. 2). In contrast to this, the parasite line containing the PF3D7_0900500 5′ *rif* ups was unable to grow in the presence of WR99210 meaning that no parasites were present which had activated this locus. Three experiments over ten growth cycles were tried with this parasite line and parasites never became resistant to WR99210. This means that the cloned PF3D7_0900500 5′ *rif* ups is either not sufficiently functional or completely silenced. The third construct containing episomes with the PF3D7_1300400 5′ *rif* ups controlling hDHFR readily grew in the presence of WR99210 and no striking difference could be discerned between blasticidin/WR99210 and blasticidin treated parasites (Fig. 2). Taken together, from the three constructs, only the integrated construct with PF3D7_0200700 5′ *rif* ups showed a dynamic that was expected for a transcriptionally variable member of the multigene family which is functional in blood stage parasites.

We then quantified the relative transcript quantity in the lineage with PF3D7_0200700 5′ *rif* ups in parasites “on” and “off” WR99210 drug pressure during the intraerythrocytic cycle. As shown in Fig. 2D and E, the relative transcript quantity difference between parasites under WR drug treatment was higher by a factor ranging from 1:4 to 1:40 using reverse transcription-qPCR in three independent experiments. The highest and significant differences were observed in trophozoite stage parasites. In order to monitor if the *rif* promotor activity could be further increased, we analyzed the steady-state transcript quantities in this parasite lineage grown under 5 nM WR99210 instead of 2.5 nM. As shown in Fig. 2E, the relative transcript quantities strongly increased under these conditions, indicating that the promoter activity may be modulated in a wide range. Importantly, the cultures that were grown in 5 nM WR99210 also did not increase the relative copy number of the artificial hDHFR, reinforcing the view that the 5′ *rif* ups PF3D7_0200700 had integrated into the genome (Supplementary Fig. 3).

**Figure 3:**
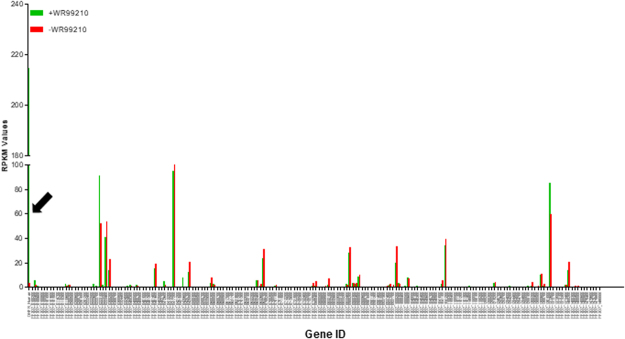
RNAseq shows no substantial differences between *rif* transcription profiles upon presence or absence of activity from a specific 5′ *rif* ups (PF3D7_0200700). A: RNAseq was conducted as described with RNA from parasites grown for four reinvasions in the presence of 2.5 nM WR99210 or in the absence of this drug. In red, the RPKM values for all *rif* loci with the presence of transcripts in parasites grown without WR99210 and in green in the presence of 2.5 nM WR99210. The arrow depicts the strongly different RPKM values for the hDHFR transcript in these cultures. Note that the difference in relative hDHFR transcript quantity is higher than measured by RT-qPCR (see Fig. 2D,E). For individual RPKM data see Supplementary Table 1.

The transcriptional activity of 5′ ups regions of *var* genes is strictly regulated resulting normally in the expression of one unique gene^33^ and silencing of all other loci, meaning that *var* loci are in crosstalk. Accordingly, it was observed that genomic *var* loci can be silenced by artificial activation of an episomal *var* 5′ upstream region^7,32^. To detect if the artificially activated *rif* locus had any influence on *rif* transcripts from other genomic loci, RNAseq was performed using a paired sample from the PF3D7_0200700 5′ *rif* ups-construct containing trophozoites which were submitted or not to WR99210 treatment. As shown in Fig. 3, parasites of this transgenic lineage showed a number of transcripts from genomic *rif* loci that appeared in higher RPKM numbers (>20 RPKM). In parasites that were not grown in the presence of WR99210, few hDHFR transcripts from the modified PF3D7_0200700 locus were detected (RPKM ~3), corroborating previous qPCR results. In contrast, higher RPKM values for hDHFR as the most abundantly detected *rif* transcripts were observed in parasites grown in the presence of WR99210. This indicates that the hDHFR-controlling *rif* promoter can be either active or inactive, pre-requisites of a variant gene promoter. Also, the RPKM values of the modified PF3D7_0200700 were in the same range as other simultaneously active *rif* promoters (e.g., PF3D7_1372600). When the relative *rif* transcript quantities from other loci were compared between parasites grown under WR99210 pressure or not, no profound changes were found. This can be interpreted that the activity of the modified PF3D7_0200700 *rif* promoter did not influence transcription from other loci. Such a result is in contrast with similar experiments using *var* promoters where the activity of a *var* 5′ ups led to a substantial silencing of the remaining genomic *var* loci^7^. When analyzing the RPKM values of all transcripts (Supplementary Table 1), a number of genes appeared differentially expressed between the two samples, and the observation that invasion-related genes were detected with higher RPKM values indicates that the sample cultivated without WR99210 was slightly advanced in the erythrocytic cycle (Supplementary Fig. 4).

**Figure 4:**
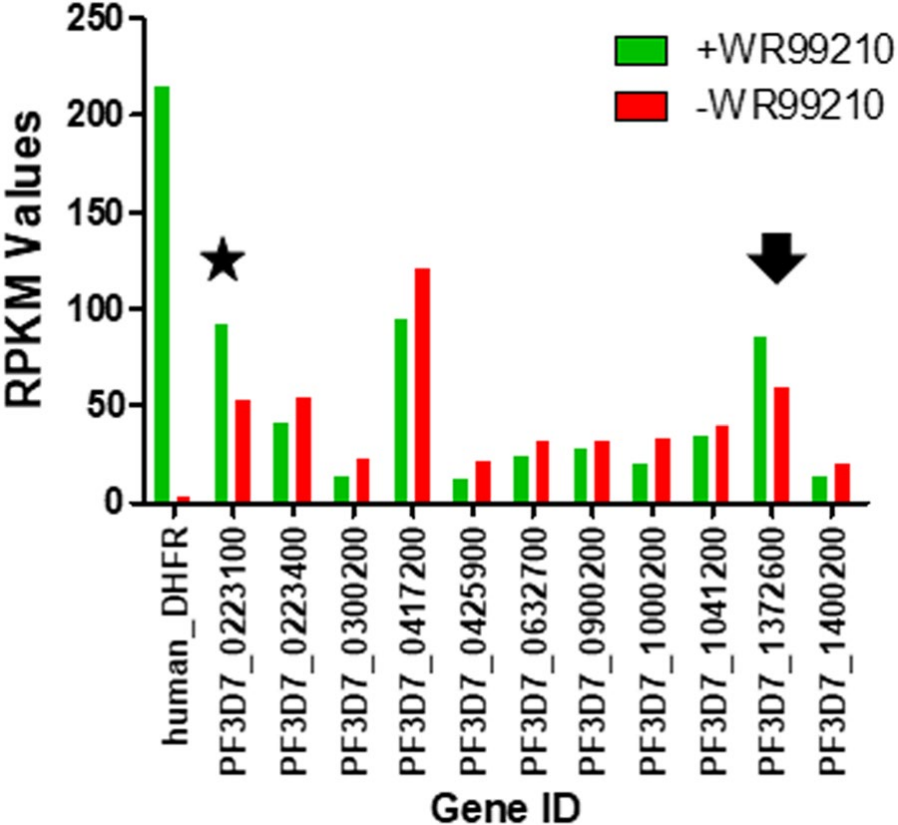
Rif loci with higher (any value above >20) RPKM values were mostly from loci with A-type rif genes. Results from Fig. 3 were filtered for higher RPKM values and are shown. Note that the locus with an almost identical 5′ *rif* ups (asterisk) on the same chromosome as the transfected 5′ *rif* ups controlling hDHFR (bars on the left) is also transcriptionally active independently of activation of the transgene 5′ *rif* ups PF3D7_0200700. The arrow indicates the only significantly transcribed type B *rif*.

It is possible that subgroups of *rif* upstream regions are regulated independently, meaning that RIFIN A and/or RIFIN B are not subject to allelic exclusion. When focusing only on *rif* transcripts which were detected in larger quantities in RNAseq (cutoff RPKMs > 20), we observed that only one *rif* B type transcript was detected in slightly elevated levels in both treated or untreated parasites, while several *rif* A-type transcripts were present (Fig. 4). This reinforces that “A”-grouped *rif* 5′ ups, encoding antigens which are potentially associated with the infected red blood cell surface, are not influenced by an artificially activated “A” type *rif* 5′-ups.

## Discussion

The expression control of variant gene families is an intriguing phenomenon in many human parasites ranging from *Trypanosoma brucei* (reviewed in^35^) and *Giardia lamblia*^36^ to *Plasmodium*. Each parasite seems to have developed different molecular mechanisms to ensure that antigenic repertoires are not prematurely exhausted. In *Giardia* trophozoites, an RNAi-based mechanism enables the translation of a single variant surface antigen per parasite^37^. In *Trypanosoma brucei*, selective variant surface antigen (VSG) production is controlled at the transcriptional level and also through genetic recombination. Single *vsg* genes are transcribed by RNA Polymerase 1 at a specific subnuclear expression site^38^ and the activation of *vsg* expression sites is associated with specific chromatin modifications (reviewed in^39^). *Var* gene expression in *P. falciparum* is tightly controlled at the transcriptional level in ring-stage parasites^40^ and all but one or two sites remain silenced. Silencing of sites is associated with a number of specific chromatin modifications, mainly at histone H3 which - when trimethylated at lysine 9 - recruits heterochromatin protein 1^12^, initially perceived as a landmark of silenced chromatin in different cell types^41^. A still not answered question is why not all *var* sites are silenced and the reason for this possibly lies in the concentration of ncRNAs transcribed from a number of GC rich regions present in the nucleus which somehow seem to coordinate gene counting^14^. Overproduction of members of these ncRNAs lead to the simultaneous transcription of *var* genes – an effect which is also observed when histone deacetylases SIR2A and SIR2B are repressed or deleted^42^. Here, we tried to interfere with the transcription of members of the *rif* gene family using an approach that was successfully applied to monitor *var* gene transcription. Interestingly, from three different constructs, only one presented a variable transcription activity, which may be considered as a pre-requisite of the corresponding RIFIN to function in antigenic variation. For the other two constructs, which also did not show integration at genomic sites, we found either a permanently activated or silenced status. Notably, all tested 5′-upstream regions have several, almost completely identical regions in the genome but only one of the *rif* genes controlled by these identical regions seemed transcriptionally active to the same degree as the transgene (not considering the 5′-ups *rif* construct with PF3D7_0900500 which was always silenced (Table 1)). Interestingly, the only identical locus with considerable amounts of transcripts from the genomic locus appeared for the differentially controlled PF3D7_0200700 *rif* 5′-ups construct. Intriguingly, the original *rif* locus PF3D7_0200700 from which the 5′-ups region was cloned had no detectable transcripts at all (Supplementary Table 1), while the *rif* locus lying on the opposite end of chromosome 2 showed transcripts in the RPKM value range of 91 and 51 depending on the presence or absence of transcripts from the artificially integrated PF3D7_0200700 *rif* locus (Table 1).

**Table 1:** Genomic *rif* 5′-ups identical to cloned *rif* 5′-ups (≥98% identity) are all but one silenced in RNAseq experiments.

5′ ups	ID	% identity	Matches	Score	RPKM	
PF3D7_0200700	PF3D7_0223100	99%	1497/1514	2936	91.36	51.95
	PF3D7_1373000	98%	1497/1520	2926	0.00	0.00
	PF3D7_0732200	99%	1491/1512	2912	0.35	0.00
	PF3D7_0101600	98%	1491/1515	2900	0.35	0.00
PF3D7_1300400	PF3D7_0600500	99%	1506/1511	2702	2.40	1.76
	PF3D7_0937500	99%	1506/1511	2702	0.00	0.00
	PF3D7_1150300	99%	1506/1511	2702	0.00	0.00
	PF3D7_0425700	99%	1502/1514	2960	0.00	0.00
	PF3D7_0100400	98%	1502/1528	2924	5.50	1.76

In the column “RPKM”, the values on the left refer to RPKM values of the WR99210 selected parasites, on the right, RPKM values of the WR99210-untreated samples are shown (without selection for hDHFR expression). Given RPKM values refer to transcripts from the *rif* genes preceded by the almost identical *rif* 5′ ups informed in the “ID” column.

This may be interpreted that absolute sequence may not be associated with promoter activity or silencing. It seems that epigenetic marking of chromatin surrounding *rif* promoters plus factors associating to them have a decisive role in 5′-ups activation and silencing, similar to what is found for *var* genes. How this regulation is achieved at the molecular level is still elusive. In the study of Howitt and colleagues, a still unknown - but limited in number- factor which provides transcriptional activation of *rif* and *var* transcripts was suggested^29^. In the present RNAseq analysis only one *var* locus showed significantly altered transcript levels (data not shown). This *var* locus – PF3D7_0400200 – is annotated as a pseudogene and consists only of exon 2. PlasmoDB predicts also the presence of the *var* intron, which itself is also a promoter. It is unclear if RNA from this site is involved in *rif* transcription regulation. Our data do not support co-regulation of *rif* and *var* genes. Of note, the material analyzed in our study may also not permit any conclusion about *rif-var* co-regulation, since RNAs from middle/late trophozoite stage parasites were employed. Normally, *var* transcription has ceased in trophozoites 20 h post reinvasion^43^. It must also be reinforced that the shown RNAseq results are unique for this specific experiment. It may be expected that the *rif* genes which appear transcribed in the current samples change over time due to switching and that in another experiment different, dominant *rif* transcripts are detected. Additionally, it is unclear if the observed *rif* transcripts occurred in the same parasites or if they originated from different parasites, concomitantly with the activated PF3D7_0200700 locus in all parasites. Also, it appears that the untreated sample was slightly advanced in the erythrocytic cycle since a number of schizont related genes such as MSPs showed more transcripts in the sample not treated with WR99210 compared to the treated sample (Supplementary Table 1, spreadsheet “2”, and “3”, Supplementary Fig. 4). Notwithstanding, the main result is still valid and other *rif* gene transcripts, besides the artificially activated PF3D7_0200700 locus, are not influenced.

Based on our data, it may be postulated the chromosomal context surrounding *rif* 5′ups-regions may play a pivotal role in *rif* expression. All of the three 5′-ups regions were designed in the same way and contained almost 1500 nt upstream of the corresponding *rif* ATG, and all three upstream regions contained either terminator regions or head-to-head promoter regions of the adjacent gene locus possibly including heterochromatin boundary regions (Supplementary Fig. 1). Nonetheless, only two 5′-ups were functional in providing a sufficient number of transcripts and only the apparently integrated allele showed a tunable behavior. In the study by Witmer and colleagues^31^, the 5′-ups region of *rif* PF3D7_1300400 was tested. This region is somehow special in that it lies in a head-to-head position to a *var* upsA promoter region. In their study, in one construct the *rif* promoter portion could be activated while the adjacent *var* ups was completely silenced. In a second, slightly modified construct, both promoters could be selected for active transcription. The degree of induction of the *rif* 5′-ups in their study was not significantly different, indicating that transcription was possibly partly induced even without drug selection for transcription from this promoter. In our study, we used 1465 nt of the same 5′ *rif* ups and also no differential regulation was observed (construct 3 with PF3D7_1300400 5′ *rif* ups). Given the ambiguous results for both *rif* 5′ ups PF3D7_0900500 and PF3D7_1300400 which are either not integrated or may have been rearranged during the transfection procedure, no further conclusions can be drawn why constructs containing these 5′ ups led to the observed results of permanent silencing or activity, respectively.

Here, the copy number of plasmids encountered in parasite lines bearing most probably episomal forms of *rif* promoted hDHFR was partially lower than in the related study from Howitt^29^ or Witmer^31^ and colleagues. This may be due to the fact that we used low concentrations of blasticidin for selection. Howitt and colleagues used 2 or 10 µg/ml blasticidin and copy numbers at 2 µg/ml were in the same range or slightly higher than in our episomal constructs. Witmer and colleagues also used 2.5 µg/ml blasticidin but their plasmid backbones contained the TARE/rep20 element, known to improve plasmid segregation^44^. When we compared results of the hDHFR marker transcript in the transgenic PF3D7_0200700 parasite line growing or not in the presence of WR99210, we found smaller discrepancies between the “on” and “off” in qPCR than in RNAseq. The reason for this may lie in the differing range of linearity of both techniques, although this was not specifically tested for.

As a central result, we showed that transcription of the *rif* gene family, specifically of the RIFIN A subset, is most probably not controlled by a mechanism related to allelic exclusion as is valid for *var* genes and that almost 100% sequence identity in *rif* 5′ ups regions is not sufficient to predict promoter co-activation or silencing. However, not all *rif* genes are transcribed meaning that there still must be some kind of controlled activation and repression. Considering the results from Guizetti and colleagues^14^, it may be postulated that ncRNAs are also involved in the activation of *rif* loci, with the difference that these ncRNAs allow for activity from multiple loci – similar to what is seen for *var* genes when certain ncRNA from GC rich regions are overexpressed^14^. From the study of Guizetti and colleagues, it is not clear what influence the *var* relevant ncRNAs had on *rif* gene transcription although these authors speculated that a co-regulation could happen. Importantly, there are ncRNAs for most *rif* loci with yet elusive function^45^. A specific transcription machinery in the periphery of the nucleus was postulated for *var* genes^46–48^. To date, it is unclear if *rif* genes are also transcribed from the same machinery. This could be elucidated by RNA FISH or other adequate methods to monitor nuclear substructures, and the created parasite line may be useful for this kind of experiment. It also still remains elusive if the B subfamily of *rif* genes is differentially regulated, given the fact that we found only one B-type *rif* gene activated in our cultures.

## Material and Methods

### Parasite culture and transfection

Parasites (strain NF54) were cultured under biological level 2 conditions at 5% hematocrit in human B+ blood supplemented with 0.5% Albumax 1 (Invitrogen) or 10% human B+ plasma in RPMI and 0.23% sodium bicarbonate under a 90% N_2_, 5% CO_2_, 5% O_2_ atmosphere or in candle jars as described earlier^49^. Human blood and plasma were obtained from the local blood bank and ethical clearance for using this blood for this research was granted by the Ethics Committee of the Institute of Biomedical Sciences at the University of São Paulo (No. 842/2016). The medium was changed daily or every two days when parasitemias were low (<0.5%). Parasitemias were checked by Giemsa-stained thin blood smears. For transfection, the protocol suggested by Hasenkamp *et al*.^50^ was used. Essentially, 150 µl of cytomix-washed fresh red blood cells were electroporated in a BioRad Gene Pulser at 310 V, 960 µF with 40 µg purified plasmid and later mixed with 2*10^7^ plasmagel-purified^51^ schizont stage parasites. On day 2 after transfection, Blasticidin (Sigma) was added at 2.5 µg/ml to the medium. At day 6 after addition of the drug, no more viable parasites were visible and the medium was changed every two days until the appearance of parasites on days 17–25 post-transfection.

### Plasmid constructs

The bi-cistronic plasmid used here is based on the pTZ57 (Thermo/Fermentas) backbone, where a cassette containing the hsp86 promoter fragment (BglII-NcoI) from pPF86^52^ was inserted. The blasticidin-deaminase coding sequence was excised from pBMNL106P-PpLuciBlast^53^ (NcoI-SalI) and inserted into the vector. Then, the hsp86 terminator sequence was inserted from pPF86 via SalI and BamHI. In this plasmid, the hDHFR resistance cassette from pRESA-GFP-HA^54^ was inserted via EcoRI/EcoRV inserting in EcoRI/SmaI. This plasmid was used to exchange the Calmodulin promoter for a 5′ upstream region from three *rif* genes which were previously shown to be transcribed in wild-type 3D7 cultures^20^. The putative *rif* promoter regions were amplified using oligonucleotides (forward/reverse) **ctcgag**atataaatttgtaaaaaccatgtg/**ggatcc**ttaattgtgatacgtatattatttaatg (precedes PF3D7_0200700, *rif* A type), **ctcgag**tattatatttttatatataattattcgtg/**ggatcc**atttaatgtgatacttatattattttatg (precedes PF3D7_0900500, *rif* B type), and **ctcgag**atgtaatatattattatgttaatattc/**ggatcc**tattgtgatacgtatattattttatg (precedes either PF3D7_1300400, PF3D7_0600500, PF3D7_0937500, PF3D7_1150300, all *rif* A type). The amplified fragments were 1400 to 1500 nt and the identity of the sequences was confirmed by semiautomatic sequencing in an Applied Biosystems 7550 sequencer. An outline of the different *rif* 5′ upstream region containing plasmid is given in Fig. 1.

### Selection of WR99210 resistant parasite lines and total RNA preparation

After outgrowth of parasites resistant to 2.5 µg/ml Blasticidin, parasites were split into two parallel cultures and cultivated in RPMI/Albumax supplemented with either 2.5 µg/ml Blasticidin/2.5 nM or 5 nM WR99210 or solely 2.5 µg/ml Blasticidin. After 4 reinvasion cycles, parasites were synchronized by plasmagel floatation^51^ and subsequent Sorbitol lysis^55^ and then harvested after another reinvasion. harvested in ring, trophozoite or schizont stage forms. Harvested IRBC were treated with 0.1% Saponin for 10 min at RT and then pelleted at 12000 g/4 °C for 5 min, washed once in 1 ml PBS and then resuspended in a volume of 100 µl using TE. Afterwards, 1 ml Trizol (Invitrogen) was added and the sample was stored at −80 °C until use. Total RNA was prepared following the Trizol protocol provided by the manufacturer. Final total RNA was dissolved in 20 µl RNAse free water and stored at −80 °C until use.

### Real-time PCR with parasite-derived cDNA and PCR test for integration

Total RNA was converted to cDNA using the previously published protocol^56^ using 5× Hotfire Pol SYBR Mix (Solis Biodyne Inc.) on an Eppendorf realplex2 thermocycler. In order to monitor the hDHFR transcription controlled by *rif* upstream regions, the following oligonucleotides were employed: forward 5′-ctggttctccattcctgagaag/reverse 5′-ttgtggaggttccttgagttct. These oligos were predicted using Primer3 online software^57^ using the same settings as used for the design of *var* oligos and the internal control oligonucleotide pair for the amplification of seryl tRNA ligase (PF3D7_0717700)^58^. Relative transcript quantities were then calculated by the 2^−ΔCt^ method^59^ using the seryl tRNA ligase transcript as endogenous control. The performance of the hDHFR oligonucleotide pair does not differ from seryl t-RNA ligase specific oligos (data not shown). To test for integration of plasmids, genomic DNAs (gDNA) were prepared from parasite cultures using the Promega genomic DNA preparation kit and aliquots of the gDNAs were tested combining forward and reverse real-time PCR oligos for hDHFR (see above) and blasticidin deaminase (forward: 5′-tgcagtttcgaatggacaaa, reverse: 5′-aacacaaaacaatctggtgcat). PCRs were performed using Thermo/Invitrogen Elongase with the following thermocycling program: 94 °C, 40 s; 54 °C, 40 s; 65 °C, 4 min, over 30 cycles.

### RNAseq with parasite-derived cDNA

Total RNA was isolated from the harvested trophozoite-stage parasites with Trizol reagent following the manufacturer’s instruction. The concentration of the isolated RNA was quantified using a Nanodrop device (Thermo Scientific, USA) and the integrity of the RNA was measured by a 2100 Bioanalyzer (Agilent Technologies, CA). Paired-end sequencing cDNA libraries were constructed from two samples (grown with or without 2.5 nM WR99210 for 6 reinvasions) using a TruSeq RNA Sample Preparation Kit v2 low sample (LS) protocol (Illumina Inc., CA), based on the manufacturer’s instructions. RNAseq was conducted in an Illumina NextSeq500 sequencer following the recommendations of the provider using mid output flow cells and a total of 10 million reads per sample. CLC Genomics Workbench 7.01 platform was used to remove the adapter and assess reads quality from the raw reads. The same platform was used to subject reads to the reference *P. falciparum* genome available in the Ensemble Genome database (Release 26) and to generate gene reads count table used in the following pipelines. Two packages built in Bioconductor^60^ were used for further analysis: edgeR^61^ and limma^62^. The first was used to filter and normalize the data set, while linear modeling and empirical Bayes to assess differential expression were performed with the limma package. Finally, the RPKM values of trophozoite-derived cDNA from cultures which were treated with WR99210 or not were loaded in MS Access and filtered for product ID containing “*rif*”, and the corresponding RPKM values for each *rif* gene were plotted against their ID.

### Southern Analysis

Genomic DNA was isolated from the WT (NF54) and NF54::pTZ57_PF3D7_0200700_ parasites, using the Wizard® Genomic DNA Purification Kit (Promega). 5 μg of each gDNA and 25ηg of plasmid DNA (pDNA) from the pTZ57_PF3D7_0200700_ construct, were digested using SpHI (Thermo Fisher Scientific). The probe was amplified using standard PCR conditions with digoxigenin-dUTP from DIG High Prime DNA Labeling and Detection Starter Kit I (Roche Diagnostics), using the Blasticidin deaminase cassette from the pTZ57_PF3D7_0200700_ construct as a template. The oligonucleotide primers used were 5′- atgggaaaaacatttaacatttc-3′ and 5′-aacacaaaacaatctggtgcat-3′. The Southern procedure was performed following the protocol provided by the manufacturer of the labeling kit (Roche), using Hybond N membranes (Amersham/GE Healthcare) and a hybridization temperature of 44 °C. All washings steps were performed at room temperature.

## Electronic supplementary material

Supplementary Figures

Supplementary Dataset 1
