# Supplementary material for: Independent regulation of Plasmodium falciparum rif gene promoters
Source: Sci Rep. 2018 Jun 19;8:9332. doi: 10.1038/s41598-018-27646-0 (PMC6008437; doi:10.1038/s41598-018-27646-0)
Supplement: Supplementary file 1 — Supplementary Figures [file 41598_2018_27646_MOESM1_ESM.pdf]

Supplementary Figures

Independent regulation of *Plasmodium falciparum rif* gene promoters

Rosana Beatriz Duque Araujo, Tatiane Macedo Silva, Charlotte Sophie Kaiser, Gabriela Fernandes Leite, Diego Alonso, Paulo Eduardo Martins Ribolla and Gerhard Wunderlich

Supplementary Figure 1

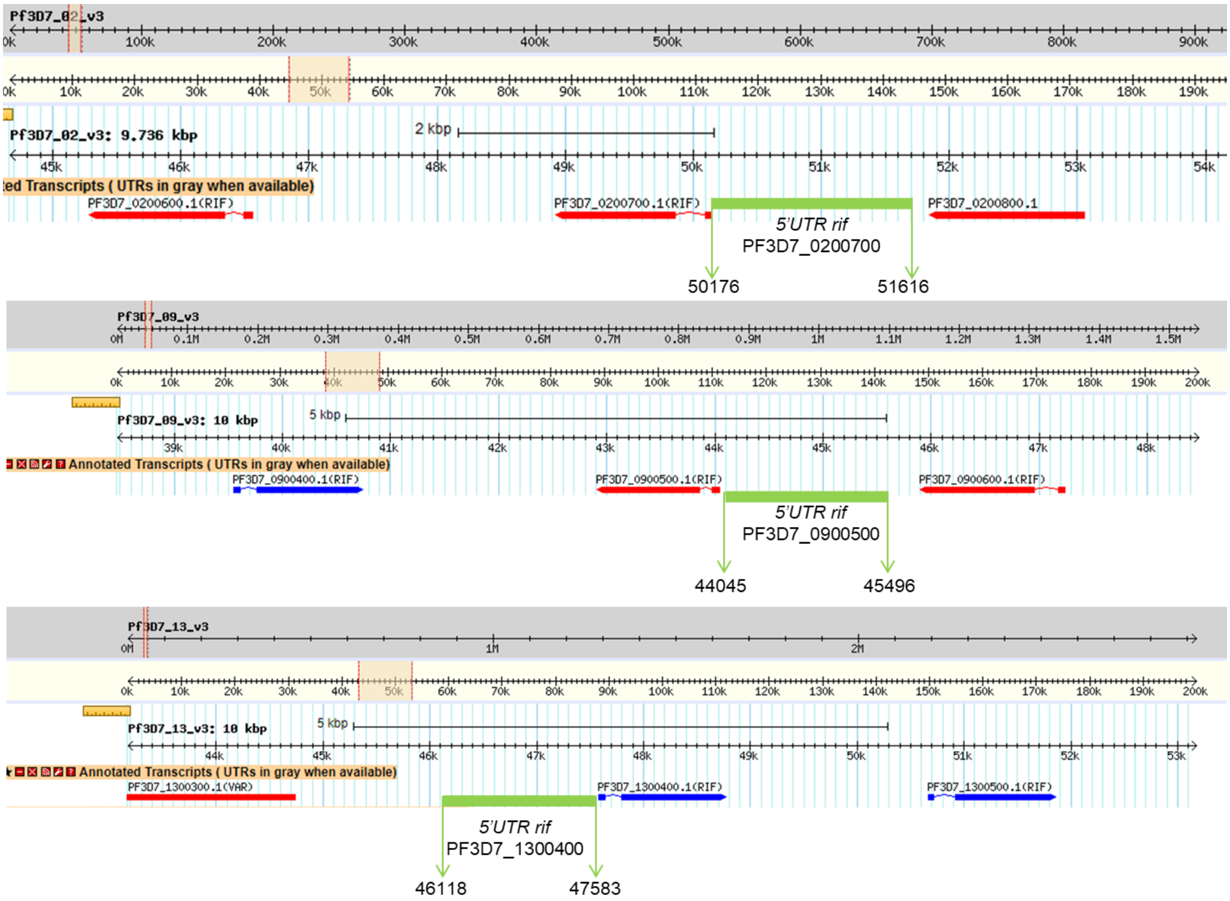

Screenshots from PlasmoDB V.36 showing the genomic context of 5‘ ups rif regions that were cloned in the three expression vectors used in the experiments.

Supplementary Figure 2

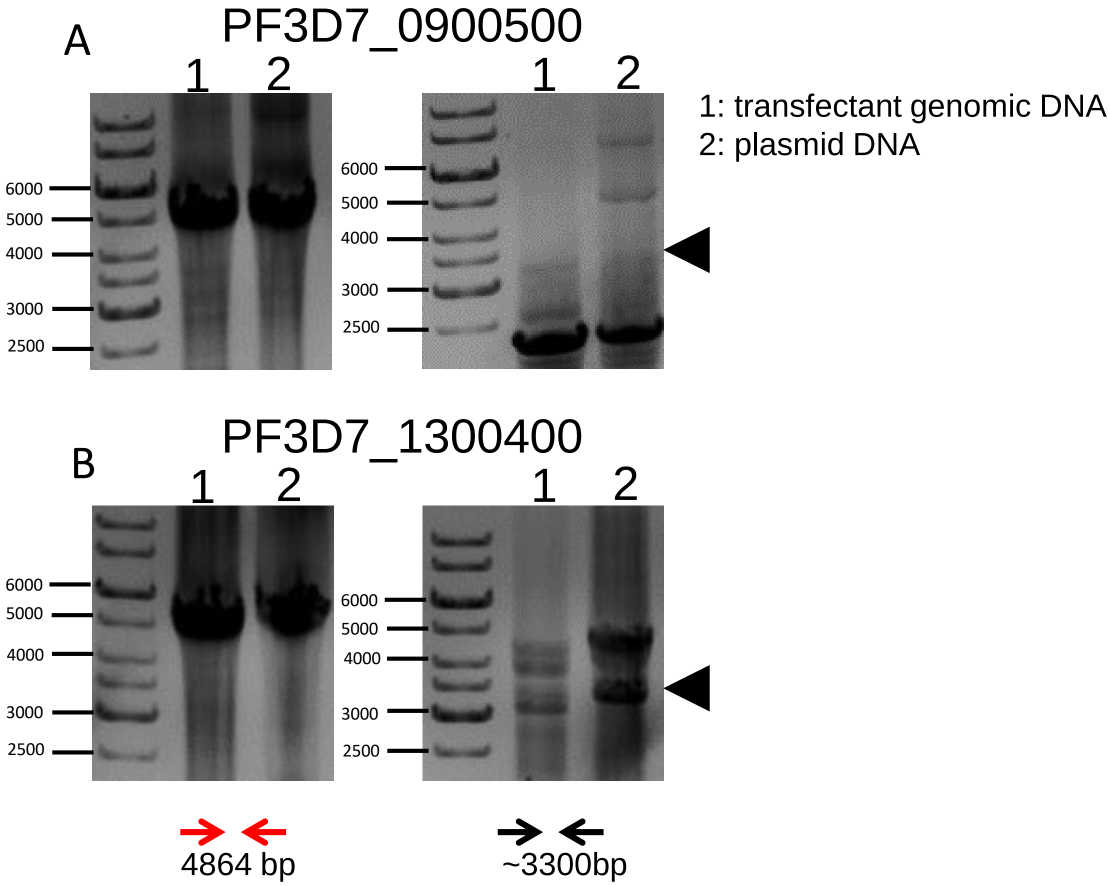

Results from PCRs using long-range polymerases show that constructs containing 5' *rif* ups PF3D7_0900500 (in **A**) and PF3D7_1300400 (in **B)** showed episomal forms in contrast to construct with PF3D7_0200700 (Figure 1). Gel pictures on the left, PCR results showing amplification with forward *hDHFR* and forward *bsd* primers (in red) over the backbone of the transfection plasmid (positive for the untransfected plasmid (2) as well as the gDNA from the 5' *rif* ups transfected strain (1)). On the right, amplification products from PCRs using reverse *hDHFR* and reverse *bsd* oligos. This amplification is only possible when the plasmid is in the episomal form (see scheme for episomal construct and integrated locus in Fig. 1 A). Probably due to the high A/T amount, amplification products over two *Plasmodium* promoter regions (see map in Figure 1) show several amplicons for the "reverse" oligo pair and the black triangle depicts the expected 3300 bp product.

Supplementary Figure 3

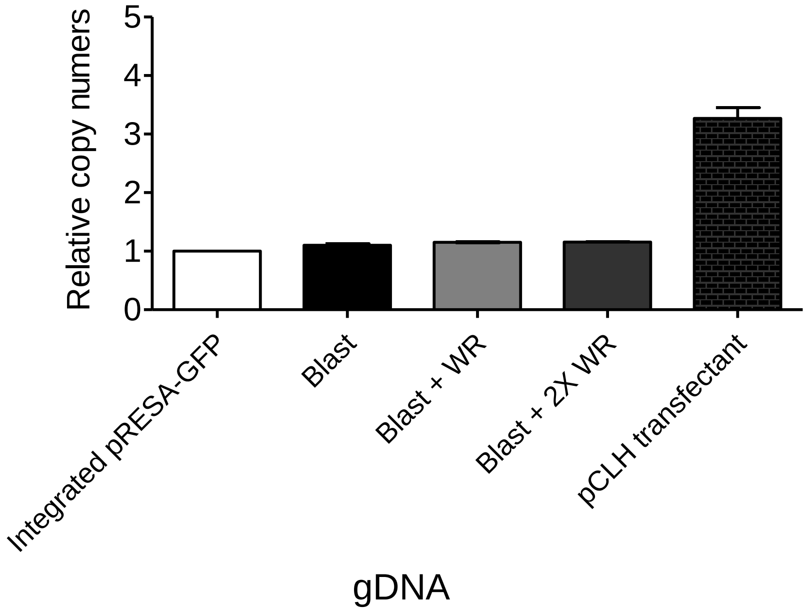

The copy number of the hDHFR locus in the PF3D7_0200700 transfectant line remains stable independent of increased drug pressure, indicating integration. As a template for qPCR using hDHFR and t-seryl synthetase oligos, genomic DNAs from indicated cultures were tested and the relative copy numbers of the hDHFR versus the genomic t-seryl synthetase locus were calculated and plotted. Note that increased presence of WR99210 did not lead to increased hDHFR copy numbers (“Blast + 2x WR” sample). WR99210 was applied in all transfectants at 2.5 nM with the exception of the “Blast + 2xWR” sample where 5 nM was used.

Supplementary Figure 4

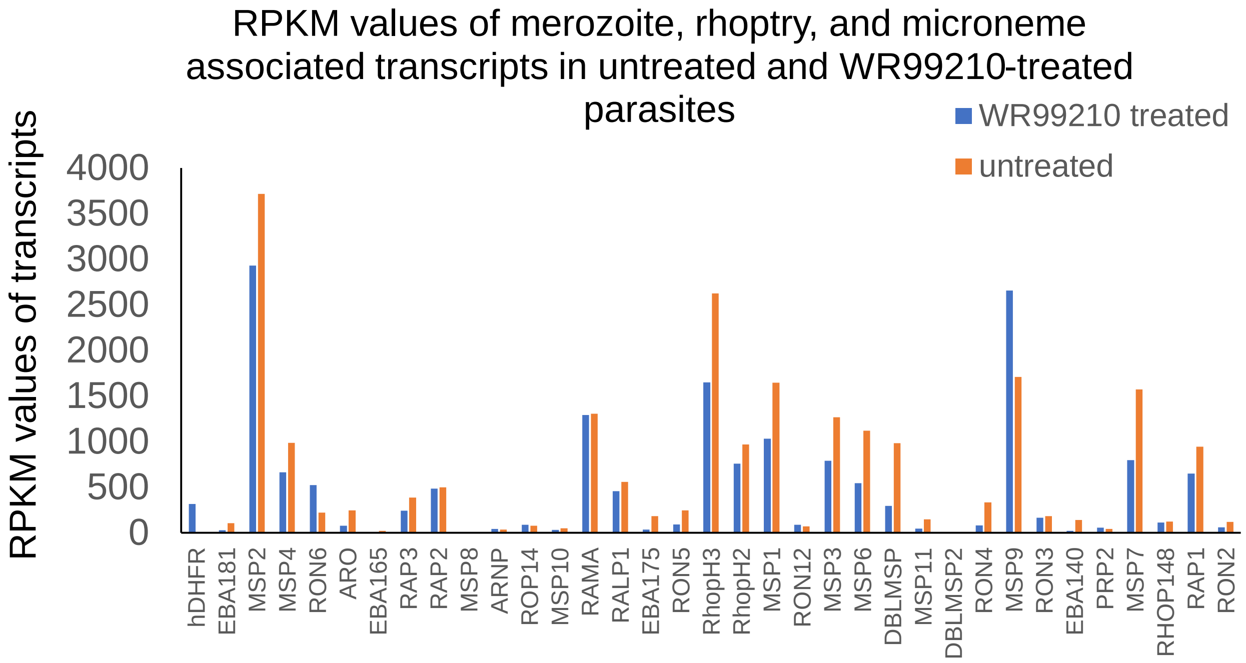

RNAseq results (RPKM values) from the parasite lineage NF54::pTZ57PF3D7_0200700 were filtered for transcripts encoding merozoite, rhoptry, and microneme proteins and plotted. Note that the untreated parasites showed higher RPKM values in typical merozoite associated factors such as MSP1 and MSP2 than the WR99210 treated parasites, indicating that untreated parasites were recovered at a slightly more advanced stage in the intraerythrocytic cycle. The hDHFR transcript from the artificially integrated rif 5‘ ups is shown on the left.

The data were extracted from Supplementary table 1 from which the corresponding PlasmoDB IDs can be obtained.

Full-length gel from Figure 1 as photographed

1: transfectant genomic DNA
2: plasmid DNA

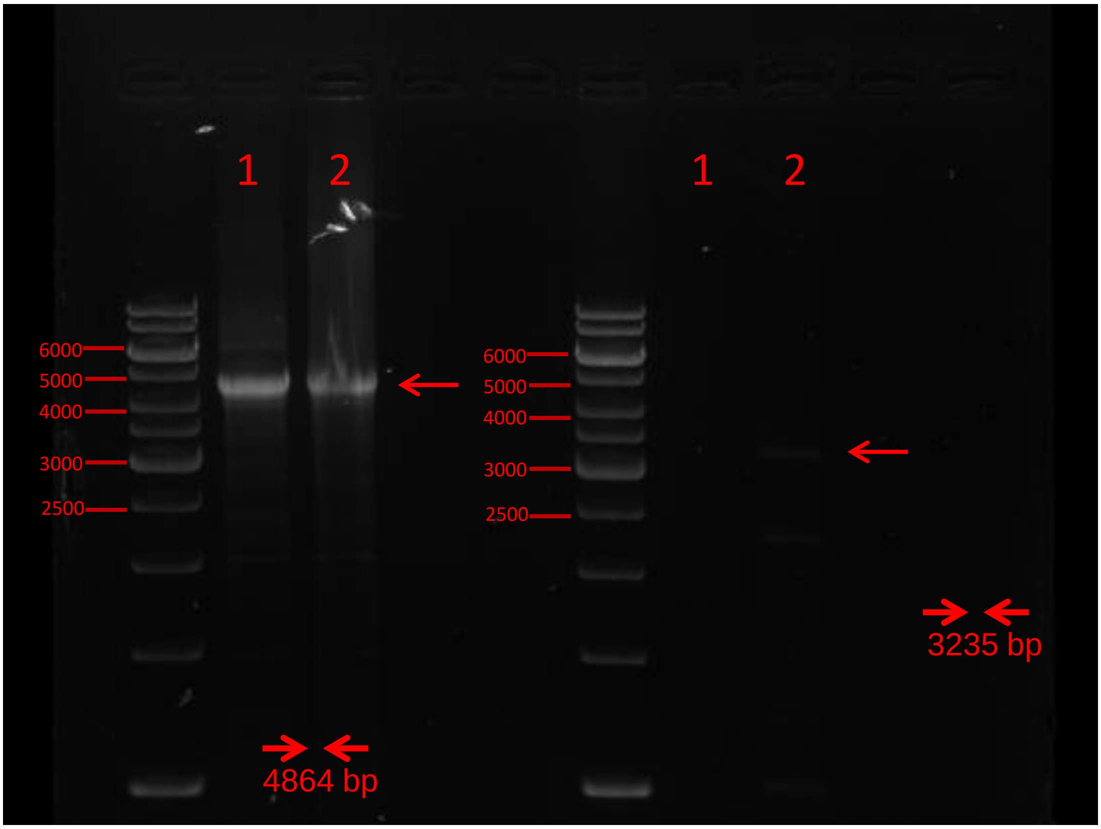

1% TAE agarose gel from Figure 1 using 1 Kb ladder from Thermo as molecular weight standard. The oligo pairs employed herein result in the indicated fragment sizes (see legend of Fig 1 for details).

Original Gel photograph from gel shown in Supplementary Figure 2 - Integration check PCR results

PF3D7_0900500 and PF3D7_1300400 5' ups constructs

1: transfectant genomic DNA
2: plasmid DNA

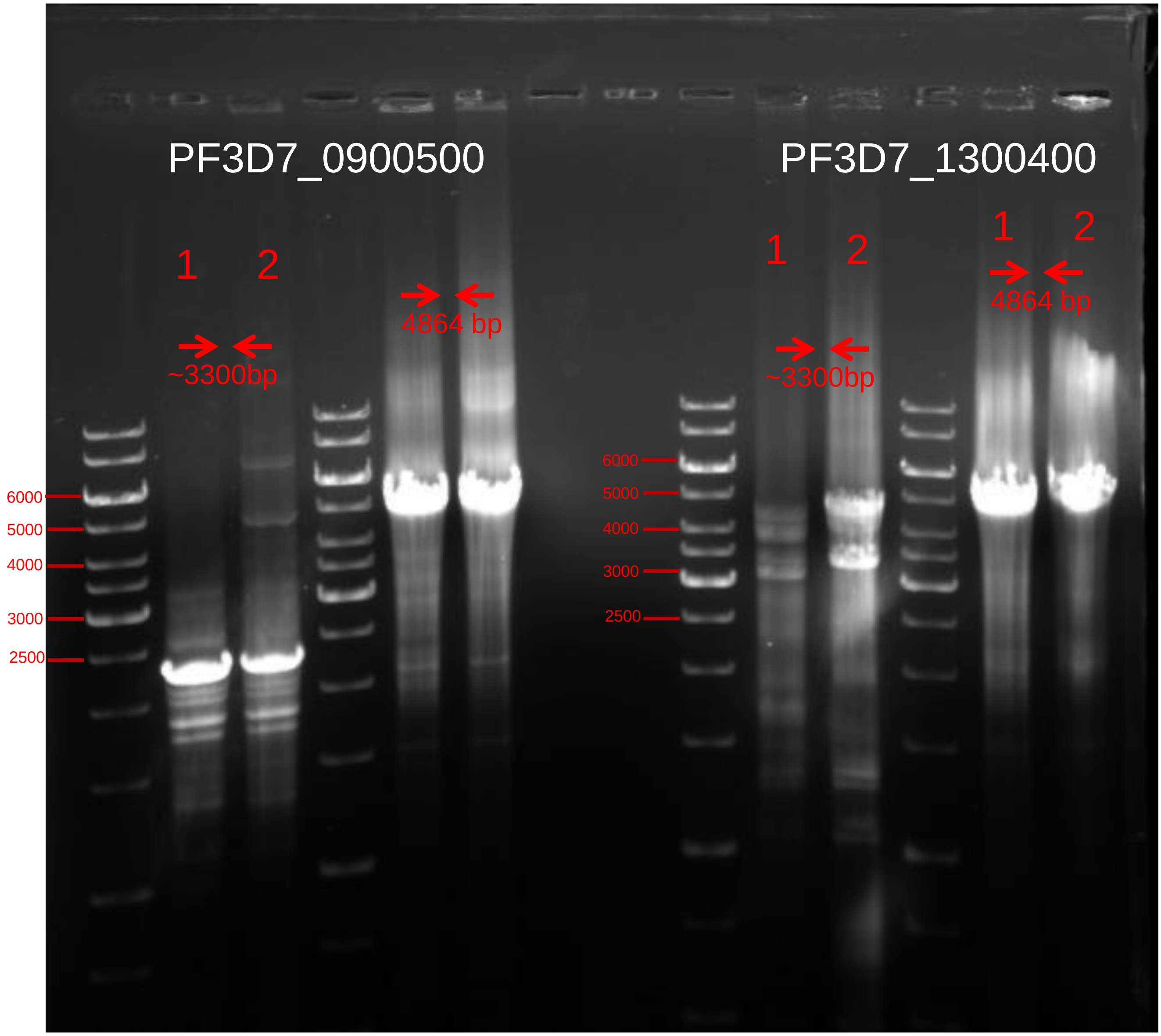

1% TAE agarose gel from Supplementary Figure 1 using 1 Kb ladder from Thermo as molecular weight standard. The oligo pairs employed herein result in the indicated fragment sizes (see legend of Supplementary Fig 1 for details).
